# Anti-inflammatory, anti-cholinergic and cytotoxic effects of *Sida rhombifolia*

**DOI:** 10.1080/13880209.2017.1285322

**Published:** 2017-02-02

**Authors:** Siau Hui Mah, Soek Sin Teh, Gwendoline Cheng Lian Ee

**Affiliations:** a School of Biosciences, Taylor’s University, Subang Jaya, Selangor, Malaysia;; b Department of Engineering and Processing, Malaysian Palm Oil Board, Kajang, Selangor, Malaysia;; c Department of Chemistry, Faculty of Science, Universiti Putra Malaysia, Serdang, Selangor, Malaysia

**Keywords:** Acetylcholinesterase, arrowleaf sida, brine shrimp, MTT, nitric oxide, protein denaturation

## Abstract

**Context:** *Sida* (Malvaceae) has been used as a traditional remedy for the treatment of diarrhoea, malarial, gastrointestinal dysentery, fevers, asthma and inflammation.

**Objectives:** This study evaluates the anti-inflammatory, cytotoxic and anti-cholinergic activities of *Sida rhombifolia* Linn. whole plant for the first time.

**Materials and methods:** *S. rhombifolia* whole plant was extracted by *n*-hexane, ethyl acetate and methanol using Soxhlet apparatus. The plant extracts were evaluated for their antioxidant (DPPH, FIC and FRAP), anti-inflammatory (NO and protein denaturation inhibitions), cytotoxic (MTT) and anti-cholinesterase (AChE) properties in a range of concentrations to obtain IC_50_ values. GC-MS analysis was carried out on the *n-*hexane extract.

**Results and discussion:** The ethyl acetate extract exhibited the most significant antioxidant activities by scavenging DPPH radicals and ferrous ions with EC_50_ of 380.5 and 263.4 μg/mL, respectively. In contrast, the *n*-hexane extract showed the strongest anti-inflammatory activity with IC_50_ of 52.16 and 146.03 μg/mL for NO and protein denaturation inhibition assays, respectively. The same extract also revealed the strongest effects in anti-cholinesterase and cytotoxic tests at the concentration of 100 μg/mL, AChE enzyme inhibition was 58.55% and human cancer cells, SNU-1 and Hep G2 inhibition was 68.52% and 47.82%, respectively. The phytochemicals present in the *n*-hexane extract are palmitic acid, linoleic acid and γ-sitosterol.

**Conclusions:** The present study revealed that the *n*-hexane extract possessed relatively high pharmacological activities in anti-inflammation, cytotoxicity and anti-cholinesterase assays. Thus, further work on the detail mechanism of the bioactive phytochemicals which contribute to the biological properties are strongly recommended.

## Introduction

Reactive oxygen and nitrogen species (ROS/RNS) are the major cause of cellular stress and result in cellular damage and DNA mutation. Antioxidant agents decrease the amount of ROS/RNS by reducing them into stabilized compounds. The elevated level of ROS/RNS in human body is dangerous and associated with the initiation and progression of cancer and inflammation (Wiseman & Halliwell 1996; Shi et al. 2012). Inflammation has long been known to contribute in the body’s defence and healing process. However, it has been proved to bring undesirable and harmful response and contribute in various diseases including asthma, rheumatoid arthritis and atherosclerosis, among others that have a high prevalence in the global population (Mueller et al. 2010). ROS/RNS have been shown to possess many characteristics of carcinogens (Cerutti 1994). Regulation of oxidative stress activity by the proteins and genes are related to cellular activities such as proliferation, differentiation and apoptosis. Oxidative damage could contribute to the deletions and mutations in mitochondrial DNA and lead to neurodegenerative diseases such as Alzheimer’s disease (Mecocci & Beal 1994).

Plants have been consumed for medicinal purposes since ancient times and have become valuable resources for primary health care systems over the centuries, especially in rural areas and developing countries (Heinrich 2010; Ullah et al. 2010). This is due to their wide availability and use as cheap therapy compared to market pharmaceuticals. Thus, the effectiveness of medicinal plant extracts against various diseases has been studied extensively worldwide. Besides, it is crucial to identify the potential candidates which play the vital roles in these pharmacological effects. Most natural extracts contain secondary metabolites, novel and structurally diverse chemical compounds, which have biologically active properties (Clark 1996). The common secondary metabolites isolated and reported to carry medicinal properties are alkaloids (Seguineau et al. 1991; Fournet et al. 1994; Ee et al. 2009), triterpenoids (Bennett et al. 1993), flavonoid (Khan et al. 2009) and coumarins (Bala & Seshadri 1971; Yasunaka et al. 2005).

*Sida rhombifolia* Linn. (Malvaceae) is known as arrowleaf sida by natives, and kurumthotti in Ayurvedic medicine. It is a short-lived perennial shrub which can grow up to 1.5 m in height. The leaves of the plants are simple, narrowly ovate to lanceolate with entire leaf blade and without foliar nectarines. Flowers are solitary, axillary with cup-shaped calyx and yellow mericarps with awns, and glabrous with free petals (Barker 1998). *Sida* spp. grows widely in tropical and hot temperate countries, particularly Malaysia and India. The plant has been widely used as traditional remedies for diarrhea, malarial, gastrointestinal dysentery, fevers, asthma and inflammation. *Sida* spp. have been proven scientifically to exhibit antibacterial (Masih et al. 2014), antioxidant (Shyur et al. 2005), anti-anxiety (Sundaraganapathy et al. 2013), anti-obesity (Thounaojam et al. 2011b), cytotoxic (Rahman et al. 2011), cardioprotective (Thounaojam et al. 2011a), nephroprotective (Thounaojam et al. 2010b) and lipid lowering (Patel et al. 2009) properties. In this study, anti-cholinesterase, anti-inflammatory and cytotoxic effects of *S. rhombifolia* whole plant are reported for the first time.

## Materials and methods

### Plant material

The whole plant of *Sida rhombifolia* was collected in November 2014 from the herb garden of Persatuan Memperbaiki Akhlak Che Ru, Endau, Johor, Malaysia. The specimen of the plant was identified by Dr. Rusea Go, Department of Biology, Faculty of Science, Universiti Putra Malaysia, where a voucher specimen no. RG5044 was deposited.

The plant sample was dried under shade and ground into fine powder and extracted successively in a Soxhlet apparatus with *n*-hexane (HEX), ethyl acetate (EtOAc) and methanol (MeOH) in sequence for 5 h each. The plant extracts were evaporated to dryness under vacuum to give HEX, EtOAc and MeOH extracts.

### Total phenolic content (TPC)

TPC of the plant extracts were performed by employing the literature methods involving the Folin–Ciocalteu reagent (Slinkard & Singleton 1977). It was carried out in a 24-well flat-bottom plates with gallic acid as a standard. Firstly, the plant extracts were oxidized by the Folin–Ciocalteu reagent. Then, the solution was neutralized by sodium bicarbonate. The mixture was allowed to stand for 2 h with intermittent shaking. The absorbance was then measured at 760 nm. The same procedure was repeated to all of the gallic acid solutions (0–1000 mg, 0.1 mL^−1^). A standard curve was constructed and the equation of *y* = 0.0026*x* + 0.0402, where *y* is absorbance and *x* is gallic acid content in μg/mL, was obtained. TPC of the plant extracts were expressed as gallic acid equivalent (μg of gallic acid/mg of crude extracts) by using the equation.

### Antioxidant assay

#### DPPH (2,2-diphenyl-1-picrylhydrazyl) radical scavenging assay

The DPPH radical scavenging assay was performed in 96-well flat bottom plates according to the methods as described by Teh et al. (2013). The DPPH solution was prepared in a concentration of 5 mg/2 mL in ethanol (EtOH). A series of concentrations, 3.13, 6.25, 12.50, 25.00, 50.00 and 100.00 μg/mL, of plant extracts were prepared in EtOH. An aliquot of 200 μL of each concentration of the plant samples was added to each well together with 20 μL of DPPH solution. Then, 200 μL of EtOH and 20 μL of DPPH solution were prepared to be used as negative control. Each sample and control was prepared in triplicates. The plate was then incubated for 30 min in dark and the absorbance of each well was recorded using a microplate reader at 517 nm. The average absorbance of each crude extract was used to determine the percentage of total radical scavenging activity by using the formula of [(A_0_− A_s_)/A_S_] × 100, where A_0_ is the absorbance of control and A_s_ is the absorbance of sample. A graph of percentage of total radical scavenging activity versus concentration was plotted to obtain the half maximal effective concentration (EC_50_) values. Three independent experiments were conducted to ensure the precision of the results. Ascorbic acid was used as the standard drug.

### Ferrous ion chelating (FIC) assay

FIC assay was conducted by the method described by Dinis et al. (1994) with minor modification. Briefly, 50 μL of 2 mM FeCl_2_ was mixed with 500 μL of each concentration of plant extract (1000, 500, 250 μg/mL), which were dissolved in methanol. The reaction was initiated by the addition of 100 μL of 5 mM ferrozine solution. The control solution comprised of all the reaction reagents without plant extract. The mixture was vigorously shaken and left to stand at room temperature for 10 min. The absorbance of the solution was thereafter measured at 595 nm. The percentage inhibition of ferrozine (Fe^2+^) complex formation was calculated as [(A_0_− A_s_)/A_S_] × 100, where A_0_ was the absorbance of the control, and A_S_ was the absorbance of the extract. Ascorbic acid was used as a positive control.

### Ferric reducing antioxidant power (FRAP) assay

FRAP assay was performed according to the method of Oyaizu (1986) with some modification. Three different concentrations 1000, 500 and 250 μg/mL of each extract (100 μL) in phosphate buffer solution (PBS) was mixed with 50 μL of PBS and 250 μL of 1% potassium ferricyanide in a centrifuge tube. Blank sample was prepared by replacing ethanol with the plant extract. Both sample and blank mixtures were incubated at 50 °C for 20 min. Then, 250 μL of 10% trichloroacetic acid was added and centrifuged at 3000 rpm for 10 min. An amount of 250 μL of supernatant was mixed with equal amount of distilled water and 50 μL of ferric chloride, and the absorbance was measured at 650 nm. Ascorbic acid was employed as the standard drug. The total antioxidant capacity of the plant extract was determined using calibration curve with an equation of *y* = 0.0011*x* + 0.1395, where *y* is absorbance and *x* is concentration of ascorbic acid (μg/mL).

### Cell culture

All the apparatus used for cell culture were sterilized and decontaminated using Hirayama HICLAVE HVE-50. Cell culture handling was carried out in an ESCO Class II BSC Biosafety Cabinet. All the cells (Source: ATCC) were incubated in 5% CO_2_ humidified incubator at 37 °C (ESCO Celculture CO_2_ Incubator with model number CCL-170B-8). Adherent cell lines cells were washed with PBS and detached with EDTA trypsin for sub-cultured after achieving confluences of 80%. Both the suspension (SNU-1 cells) and adherent (RAW264.7 and Hep G2 cells) healthy cells were spun down to separate healthy cells from unhealthy and dead cells by using Thermo Scientific (Langenselbold, Germany) Sorvall ST 16R centrifuge machine at 1800 rpm for 5 min. All the cell stocks were placed in an −86 °C ultra-low freezer and preserved in a liquid nitrogen tank (Taylor-Wharton, Mildstedt, Germany LS300).

### Anti-inflammatory assay

#### Nitric oxide (NO) assay

NO assay was performed with the Griess reagent as described previously (Ee et al. 2011). Raw cells (2 × 10^6^ cells/mL) were cultivated in DMEM supplemented with 10% FBS. The cells were seeded in a 96-well plate and incubated for 2 h. The plant extracts were dissolved in DMSO and serially diluted to concentrations ranging of 100, 50, 25, 12.5, 6.25 and 3.13 μg/mL by medium. The cells were then induced with 10 μg/mL of lipopolysaccharide (LPS) in the presence of plant extracts and made up to a final volume of 100 μL. The plate was incubated further for 24 h. A fresh culture medium was used as blank. The supernatant of LPS-induced RAW 264.7 cell cultures was mixed with an equal volume of Griess reagent and incubated for 10 min at room temperature. The absorbance was taken at OD = 550 nm. All determinations were performed in triplicate and expressed as mean ± S.E.M. The standard drug used in this assay was diclofenac sodium.

#### Protein denaturation assay

Protein denaturation assay was carried out according to the previous reported methods by Mizushima and Kobayashi (1968) with minor modification. The 5 mL of reaction mixture consisted of 0.2 mL of egg albumin, 2.8 mL of phosphate buffered saline (pH 6.4) and 2 mL of varying concentrations of plant extracts to make up the final concentrations of 31.25, 62.50, 125.00, 250.00 and 500.00 μg/mL. Similar volume of PBS served as negative control. The mixtures were then incubated in a BOD incubator at 37 °C for 15 min and heated at 70 °C for 5 min. After cooling, their absorbance was measured at 660 nm by using vehicle as blank. Diclofenac sodium was used as a reference drug. The inhibition of protein denaturation was calculated by using the following formula:

Percentage of inhibition =[(C-B)-(A-B)]/(A-B)×100]

where A = average of absorbance of negative control

B = average of absorbance of blank

C = average of absorbance of sample

#### Anti-proliferative (MTT assay)

The 3-(4,5-dimethylthiazol-2-yl)-2,5-diphenyltetrazolium bromide (MTT) assay was performed according to Mosmann (1983) in sterile 96-well flat bottom plates. Both SNU-1 and Hep G2 cells (2 × 10^6^ cells/mL) were cultivated in RPMI media with 10% FBS. The concentration of plant extracts used was 100 μg/mL for both cell lines. Each plant extract was tested in triplicate together with the controls. After incubation at 37 °C and 5% of CO_2_ for 72 h, 20 μL of MTT solution was added into each well and incubated further for 3 h. The plate was spun at 1500 rpm for 10 min. This is followed by discarding approximately 80% of the supernatant and the equal volume of DMSO was replaced. The absorbance was determined at OD = 550 nm after the purple crystal formazan was fully dissolved in DMSO. Three independent experiments for both cell lines were conducted. The average absorbance values were used in the calculation of percentage cell viability.

#### Brine shrimp lethality assay

A brine shrimp hatchery kit was used to culture and hatch the eggs of the shrimp, *Artemia salina*. The conical shaped vessel was filled with sterile artificial seawater (pH 8.5) under constant aeration and light for 48 h. After hatching, ten nauplii were drawn and placed into culture plate with 6 mL of brine solution. The plant extracts with different concentration in methanol were added into the plate and incubated at room temperature for 72 h under light and surviving larvae were counted. The percentage of lethality was determined and LC_50_ values were obtained from the graph of concentration versus percentage of lethality. Three independent experiments were conducted to ensure the precision of the results. Podophyllotoxin was used as the standard drug.

#### Anti-cholinesterase assay

Acetylcholinesterase (AChE) inhibitory activities were performed according to Ellman et al. (1961) with some modification by using electric eel AChE. Acetylthiocholine iodide was employed as a substrate of the reaction while DTNB [5,5′-dithio-bis(2-nitrobenzoic acid)] was used for the measurement of the cholinesterase activity. Briefly, 150 μL of 100 mM sodium phosphate buffer (pH 8.0), 10 μL of 0.5 mM DTNB, 10 μL of plant extract (100 μg/mL) in ethanol and 20 μL AChE (5.32 × 10^−3^ U) solution were added into a 96-well plate and incubated for 15 min at 25 °C. The reaction was then initiated by the addition of 10 μL of acetylthiocholine iodide (0.71 mM). The hydrolysis of these substrates was observed by the formation of yellow 5-thio-2-nitrobenzoate anion as the result of the reaction of DTNB with thiocholine, and the absorbance is taken at 412 nm. The experiments were carried out in triplicate. Tacrine was used as a standard compound. Percentage of inhibition of AChE or BChE was determined against blank sample (sodium phosphate buffer pH 8) using the formula, (E − S)/E × 100, where E is the activity of enzyme without test sample and S is the activity of enzyme with test sample.

#### Gas chromatography-mass spectrometry (GC-MS) analysis

The hexane extract was analyzed by gas chromatography equipped with mass spectrometry (GC-MS-QP2010 Plus-Shimadzu, Tokyo, Japan). The column temperature was set to 50 °C for 1 min, increased to 200 °C at the rate of 30 °C/min, and increased to 300 °C at the rate of 15 °C/min, and then held for 25 min. The injector temperature was set at 280 °C (splitless mode, injection volume = 0.1 μL). Total run time was 25 min. Mass spectra were obtained from the range *m*/*z* 35 to 750 and the electron ionization at 70 eV. The chromatograms of the sample were identified by comparing their mass spectra with NIST08 library data, and the GC retention time against known standards.

### Statistical analysis

The *t*-test and one-way ANOVA were used to determine the statistical significance of differences between the values for the various experimental and control groups. Data are expressed as means ± standard deviation (S.D.) and the results are taken from at least three independent experiments performed in triplicate. The *p*-values of 0.05 or less were considered statistically significant.

## Results

### Total phenolic content (TPC)

The total phenolic contents (TPC) of crude extracts were performed by employing the methods involving the Folin–Ciocalteu reagent with gallic acid as a standard (Slinkard & Singleton 1977) and the results are presented in Table 1. The EtOAc extract of *S. rhombifolia* exhibited highest total phenolic contents with 72.14 μg of GA/mg of extract. It is followed by the MeOH extract with 67.62 GA/mg of extract.

**Table 1.** The antioxidant properties and total phenolic contents of *Sida rhombifolia*.

	EC_50_ (μg/mL)			
Plant Extract	DPPH Assay	FIC Assay	Total Antioxidant Capacity(μg of ascorbic acid equivalent/mg of extracts)	Total Phenolic Content (μg of gallic acid equivalent/mg of extracts)
HEX	>800.0	558.2 ± 2.3^a^	695.4 ± 1.5^a^	ND
EtOAc	380.5 ± 2.8^a^	263.4 ± 3.5^b^	736.5 ± 1.9^b^	72.14 ± 6.39
MeOH	726.2 ± 2.6^b^	577.6 ± 3.0^c^	384.5 ± 1.1^c^	67.62 ± 2.59
Ascorbic Acid	31.3 ± 1.3	40.0 ± 1.3	–	–

The values are expressed as mean ± SD in triplicate. Means with different letters are siginificantly different (*p* < 0.05). ND: Not detected; ?: not tested.

### Antioxidant assay

#### DPPH (2,2-diphenyl-1- picrylhydrazyl) radical scavenging assay

**Figure 1.**
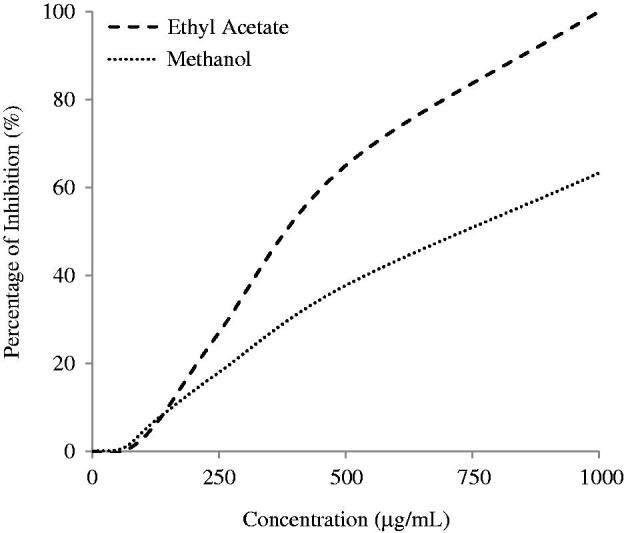
DPPH radical scavenging activity of *S. rhombifolia.* Each data point represents the mean ± SD of three independent experiments.

The DPPH radical scavenging assay is a widely use method to evaluate the antioxidant activities of the plant extracts and phytochemicals. The EC_50_ values of the plant extracts are tabulated in Table 1. Figure 1 shows that the EtOAc extract exhibited the highest scavenging activity against DPPH radicals with an EC_50_ of 380.5 μg/mL. It is followed by MeOH extract with higher EC_50_ of 726.2 μg/mL. However, the HEX extract did not show significant scavenging effect within the range of tested concentration.

### Ferrous ion chelating (FIC) assay

**Figure 2.**
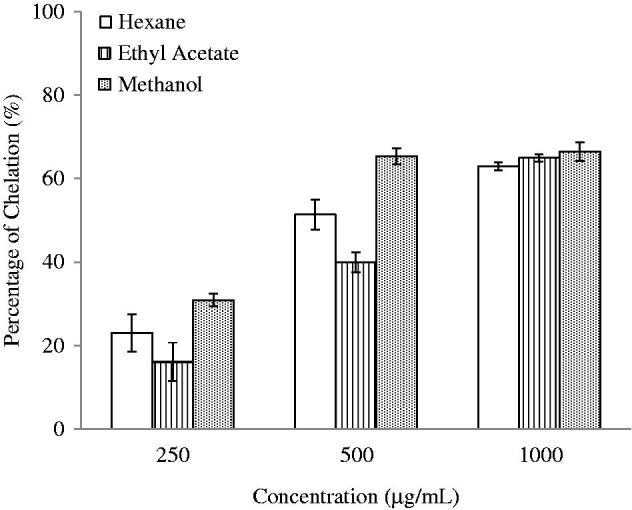
Ferrous ion chelating capability of *S. rhombifolia*. Each data point represents the mean ± SD of three independent experiments. Bars denote statistically significant differences at *p* < 0.05.

The FIC assay was carried out to assess the chelation capacity of the plant extracts (Figure 2). The results in Table 1 illustrated that the EtOAc extract of *S. rhombifolia* possessed remarkable chelation power with EC_50_ of 263.4 μg/mL. The HEX and MeOH extracts (EC_50_ of 558.2 and 577.6 μg/mL, respectively) show weaker chelation power if compared to EtOAc extract.

### Ferric reducing antioxidant power (FRAP) assay

**Figure 3.**
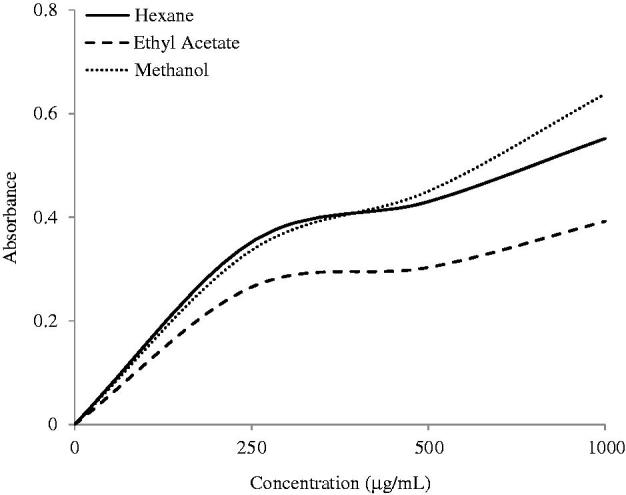
Ferric-reducing antioxidant power of *S. rhombifolia*. Each data point represents the mean ± SD of three independent experiments.

The FRAP of the plant extracts were determined by the calibration curve of ascorbic acid and the results were presented in Table 1. The graphs that are shown in Figure 3 demonstrated that the total antioxidant capacities of plants are in the concentration-dependent manner. The HEX and MeOH extracts of *S. rhombifolia* exhibited comparable total antioxidant capacities with 558.2 and 557.6 μg of AA equivalent/mg of extracts. On the other hand, the EtOAc extract showed weaker total antioxidant capacity at 263.4 μg of AA equivalent/mg of extract.

## Anti-inflammatory assay

### Nitric oxide (NO) assay

**Figure 4.**
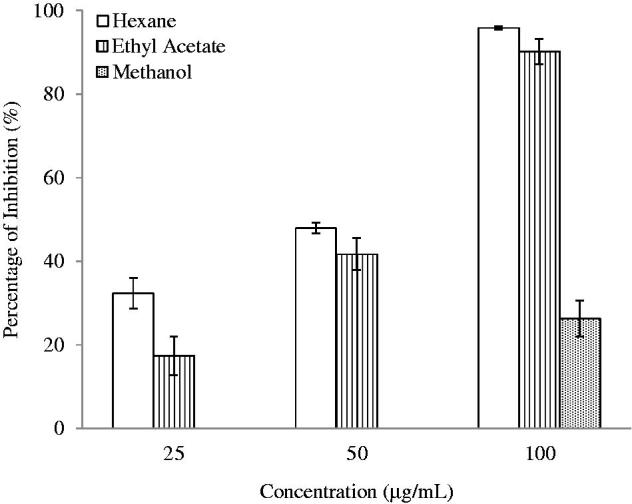
Anti-inflammatory activity of *S. rhombifolia* on NO Inhibition. Each data point represents the mean ± SD of three independent experiments. Bars denote statistically significant differences at *p* < 0.05.

**Figure 5.**
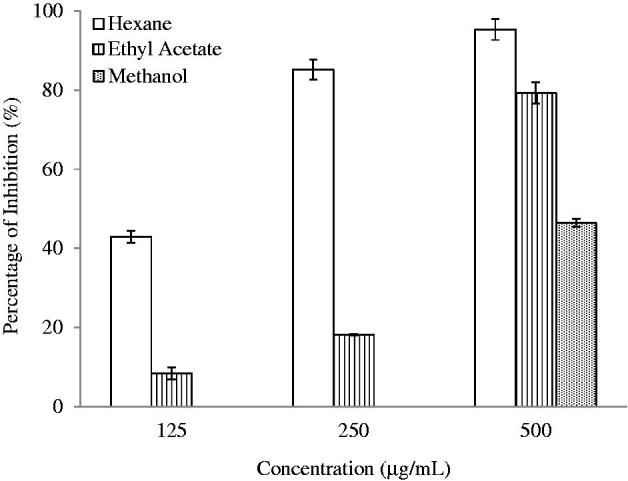
Anti-inflammatory effect of *S. rhombifolia* on protein denaturation. Each data point represents the mean ± SD of three independent experiments. Bars denote statistically significant differences at *p* < 0.05.

The NO inhibition effects of plant extracts on LPS-induced RAW 264.7 cells were evaluated and summarized in Table 2. The inhibition of NO production obtained for the plant extracts were in concentration-dependent manners as shown in Figure 5. Both the HEX and EtOAc extracts of *S. rhombifolia* showed positive results in this assay with IC_50_ of 52.16 and 58.57 μg/mL, respectively. On the other hand, the IC_50_ value of MeOH extract is not detected with the range of tested concentrations and the inhibition activity of MeOH extract can only be observed at the concentration of 100 μg/mL as shown in Figure 4.

**Table 2.** Anti-inflammatory properties of *Sida rhombifolia*.

	IC_50_ (μg/mL)	
Plant Extract	NO Assay	Protein Denaturation Assay
Hex	52.16 ± 2.40^a^	146.03 ± 2.32^a^
EtOAc	58.57 ± 4.06^a^	380.29 ± 0.90^b^
MeOH	>100.00	>500.00
Diclofenac sodium	5.02 ± 0.38	304.72 ± 9.08

The values are expressed as mean ± SD in triplicate. Means with different letters are siginificantly different (*p* < 0.05).

### Protein denaturation assay

The results of protein denaturation assay for the evaluation of anti-inflammatory properties of plant extracts are similar to that of NO assay as presented in Table 2. For this assay, the HEX extract exhibited potent inhibition towards protein denaturation with a low IC_50_ of 146.03 μg/mL. The protein denaturation activity possessed by HEX extract is stronger than the standard drug, diclofenac sodium. Besides, the EtOAc extract demonstrated comparable inhibition activities with diclofenac sodium (IC_50_ 380.29 μg/mL). Similarly, the inhibition activity of MeOH extract is much weaker and detected only at the concentration of 500 μg/mL as shown in Figure 5.

### Anti-proliferative (MTT assay)

**Figure 6.**
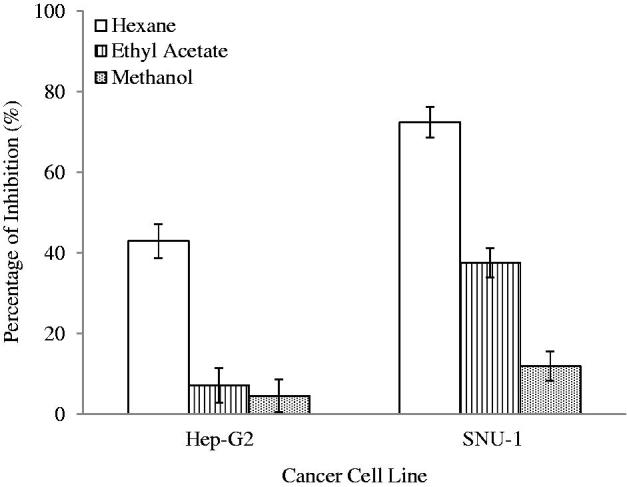
Anti-proliferative activity of *S. rhombifolia* at 100 μg/mL against cancer cells. Each data point represents the mean ± SD of three independent experiments. Bars denote statistically significant differences at *p* < 0.05.

The human cancer cell lines used in the evaluation of anti-proliferative effects of plant extracts were Hep G2 (liver cancer) and SNU-1 (stomach cancer). The cytotoxicity of the plant extracts evaluated at the concentration of 100 μg/mL are presented in Figure 6. The results show that the crude extracts of *S. rhombifolia* exhibited stronger cytotoxic effects against SNU-1 cells. The HEX extract of *S. rhombifolia* showed the strongest cytotoxicity in both cell lines. It was able to inhibit 68.52% and 47.82% of the SNU-1 and Hep G2 cells, respectively. The EtOAc and MeOH extracts exhibited weaker cytotoxic effects. The EtOAc extract inhibited 33.74% of SNU-1 cells and 3.61% of Hep G2 cells.

### Brine shrimp lethality assay

**Figure 7.**
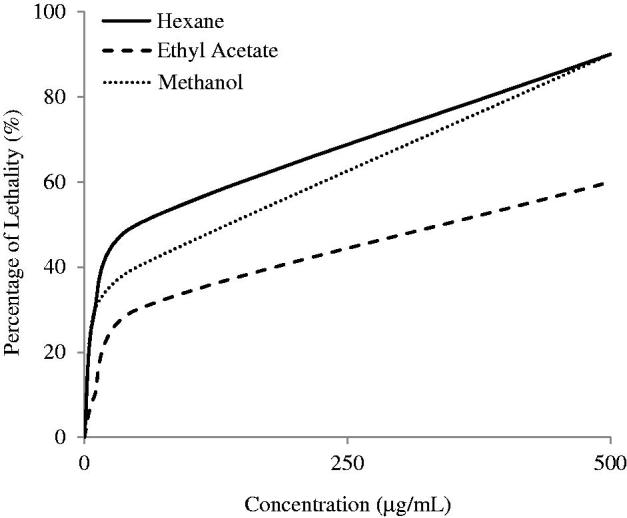
The lethality of *S. rhombifolia.* Each data point represents the mean ± SD of three independent experiments.

Brine shrimp assay was performed by using *Artemia salina* and the LC_50_ values were obtained from Figure 7. The most toxic plant extract is HEX extract with an LC_50_ value of 40.95 μg/mL. The EtOAc and MeOH exhibited moderate lethality with LC_50_ of 231.69 and 138.30 μg/mL, respectively.

### Anti-cholinesterase assay

**Figure 8.**
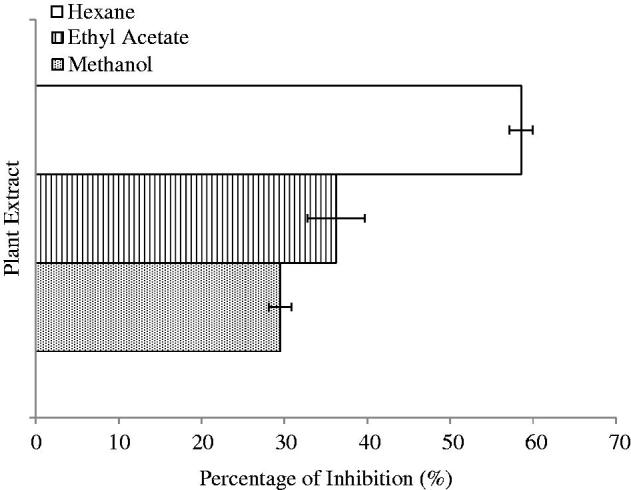
Anti-cholinesterase Activity of *S. rhombifolia* at 100 μg/mL. Each data point represents the mean ± SD of three independent experiments. Bars denote statistically significant differences at *p* < 0.05.

For anti-cholinesterase assay, AChE enzyme and acetylthiocholine iodide (ATCI) substrate were used and the hydrolysis of ATCI was monitored and measured as the inhibition effect of AChE enzyme. The results are shown in Figure 8 as the percentage of enzyme inhibition at the concentration of 100 μg/mL. It demonstrated that the HEX extract of *S. rhombifolia* possessed the highest inhibition at 58.55%, and followed by EtOAc (36.23%) and MeOH (29.48%) extracts.

### Gas chromatography-mass spectrometry (GC-MS) analysis

**Figure 9.**
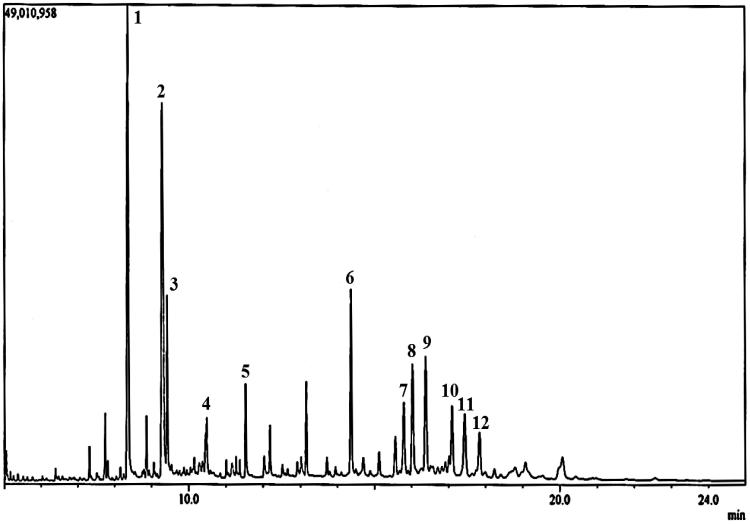
Gas chromatography spectrum of HEX extract of *S. rhombifolia*.

A GC-MS analysis was performed on the HEX extract of *S. rhombifolia,* which exhibited strongest activity in anti-inflammatory, anti-proliferative and anti-cholinergic compared to the EtOAc and MeOH extracts. Twelve chemical constituents were identified from the chromatogram as shown in Figure 9. The major chemical constituents present are palmitic acid (**1**), linoleic acid (**2**), tetracontane (**6**) and γ-sitosterol (**9**) with relative peak areas of 15.86, 15.44, 5.38 and 5.00%, respectively (Table 3).

**Table 3.** Phytochemical constituents identified in the HEX extract of *Sida rhombifolia* by GC-MS analysis.

Peak	Compound	Retention time (min)	Peak area (%)
1	Palmitic acid	8.369	15.86
2	Linoleic acid	9.290	15.44
3	Octadecanoic acid	9.421	4.36
4	Eicosanoic acid	10.483	2.16
5	Docosanoic acid	11.529	2.11
6	Tetracontane	14.368	5.38
7	Stigmasterol	15.790	2.98
8	Tetrapentacontane	16.028	4.68
9	γ-Sitosterol	16.383	5.00
10	Lupenone	17.094	2.89
11	Lupeol	17.438	3.51
12	Sitostenone	17.835	2.03

## Discussion

In the present study, several experiments were used to investigate the correlation between total phenolic content and antioxidant properties. Overall, the EtOAc extract of *S. rhombifolia* exhibited the strongest antioxidant properties in DPPH, FIC and FRAP experiments, whereas the MeOH extract exerted mild scavenging activity. The results are in agreement with the previous study done by Konaté et al. (2010) on the whole plant extracts of *S. alba* and *S. acuta*. Furthermore, the antioxidant results are positively correlated with the total phenolic content of the plant extracts. DPPH radical assay is widely used to evaluate the free radical scavenging ability of potential agents. A study reported in 2010 revealed an EC_50_ of 63.23 μg/mL for DPPH scavenging effect of methanol leaf extract of the same plant, which was collected from North East India (Thounaojam et al. 2010a). However, the EC_50_ of the whole plant methanol extract that was obtained in this study is 726.2 μg/mL. This shows that the leaf extract of *S. rhombifolia* is more likely to possess stronger DPPH scavenging effects. In comparison with the DPPH scavenging effects of the whole plants, Dhalwal et al. (2007) reported that the ethanol extract demonstrated an EC_50_ of 983.8 μg/mL, which has a weaker effect if compared to EtOAc (380.5 μg/mL) and MeOH (726.2 μg/mL) extracts in this study. The positive results obtained indicated that these extracts are able to stabilize the free radicals by transferring an electron or hydrogen atom to DPPH (Oyaizu 1986).

The FIC result of the whole plant of *S. rhombifolia* was reported for the first time with an EC_50_ of 263.4 μg/mL. The ion chelating properties is important due to the accumulation of transition metal ions causes tissue damage and leads to cancer, inflammation, and Alzheimer’s disease (Ercal et al. 2001). Thus, the FIC results highlight the therapeutic importance of the chelation capacities of the EtOAc extract of *S. rhombifolia*. Similar to DPPH scavenging effects, the metal chelating effect of methanol extract of whole plant gave lower effects than the methanol leaf extract (EC_50_ 65.69 μg/mL), described by Thounaojam et al. (2010a). Apart from the difference in part of the plant used, the huge difference observed for both DPPH and FIC results might be due to geographical location of the plant, which results in the deviation of biological activities (Kujumgiev et al. 1999; Banerjee & Bonde 2011). Previous studies revealed that the ion-chelating capacity is correlated to the electron-donating ability (Aruoma 2003; Roginsky & Lissi 2005), suggesting that the electron-donating ability attributed to the observed overall antioxidant properties. Consequently, the antioxidant result of the plant extracts of *S. rhombifolia* observed in this study supported its traditional use for various types of oxidative stress diseases (Nadkarni 1982), which are associated with the initiation and progression of inflammation and cancer (Wiseman & Halliwell 1996; Shi et al. 2012).

Lipopolysaccharide (LPS) activates and stimulates macrophage and resulting in the secretion of pro-inflammatory cytokines such as inducible NO synthase (iNOS) during inflammation (Zhou et al. 2008). The production of NO will lead to inflammation and immunoregulation (Ialenti et al. 1992). On the other hand, protein denaturation is one of the causes of inflammation and arthritic diseases (Opie 1962). The anti-inflammatory properties of plant extracts of *S. rhombifolia* were evaluated by NO production inhibition effects in LPS-induced responses in macrophage cell line, RAW 264.7, in addition to protein denaturation assay and the results are reported for the first time. Previous studies on the anti-inflammatory properties of this plant were done on animal models and the plant extracts were reported to show oedema suppressant activity (Kumar & Mishra 1997; Venkatesh et al. 1999). In the present study, the HEX extract of *S. rhombifolia* inhibited the secretion of NO significantly and exhibited strongest protein denaturation properties among the plant extracts. The results from both assays are consistent and these activities of the plant extracts are observed to be polarity dependent, at which the non-polar extract possessed stronger activities. This is supported by a previous study on anti-inflammatory study by using the carrageenin-induced oedema in rat paw, which showed that HEX leaf extract of *S. rhombifolia* showed stronger effect than the EtOAc and MeOH extracts (Venkatesh et al. 1999). The percentage inhibition of oedema by HEX leaf extract reported was 20.9% at the dose of 200 mg/kg. The anti-inflammatory activities of the plant extracts of *S. rhombifolia* shown in current study supported the traditional usage of this plant by native people in the treatments or prevention of rheumatism, inflammations and pain-related illness (Nadkarni 1982).

Cytotoxic assay is extensively used to evaluate the cytotoxic effects of the medicinal plants towards cancer cells. The cytotoxic effects of the plant extracts towards Hep-G2 and SNU-1 cells were also observed to be polarity dependent. The effects are seen to be increased with a decrease in the polarity of the plant. The cytotoxic effect of *S. rhombifolia* towards SNU-1 cells is reported for the first time and the effects shown were relatively higher than that of Hep G2 cells. The MeOH extract, which possessed the weakest cytotoxicity against Hep G2 cells in this study, was reported previously to give an IC_50_ of 475.33 μg/mL (Pieme et al. 2010). Our present study discovered that the HEX extract exhibited stronger cytotoxicity than the MeOH extract, thus revealing that HEX extract is definitely contributing an even lower IC_50_. The cytotoxic effects of *S. rhombifolia* were further confirmed by the toxicity assay by using brine shrimp, where the HEX extract appeared to be the most toxic among the extracts. The HEX extract had an LC_50_ of 40.95 μg/mL, which is similar to that of polar ethanol extract of the aerial part of this plant (Rahman et al. 2011). However, the toxicity of polar MeOH was shown to be three times weaker. This indicates that the ethanol extract of aerial part is highly toxic if compared to the MeOH extract of whole plant, even though the polarities of ethanol and MeOH extracts are similar. The explanation could be the difference in phytochemicals present in the root and aerial parts of the same plant. Besides, toxicity of the plant extracts of *S. rhombifolia* were evaluated in mice and rat models and the results suggested that these plant extracts are safe for oral consumptions up to 2000 mg/kg (Sireeratawong et al. 2008; Sarangi et al. 2011). The cytotoxicity of these plant extracts may be contributed by the bioactive alkaloids, such as vasicinol, ephedrine, vasicinone and hypaphorine, which have been isolated from *Sida* plants previously (Prakash et al. 1981; Chaves et al. 2013). The findings of *in vitro* cytotoxic effects against cancer cells with low toxicities *in vivo* reveal the potential of the plant extracts of *S. rhombifolia* to be further studied and developed into pharmaceutical agents.

Alzheimer’s disease (AD) is an irreversible degeneration of cholinergic neurons disease, which affects mainly the elderly people. The neurodegenerative effect is due to the rapid hydrolysis by the enzyme of acetylcholinesterase (AChE), which decreases the level of acetylcholine (Bartus et al. 1982). Thus, the treatment of AD is based on the inhibition of the AChE in order to improve the cholinergic neurotransmission and extensive research on the discovery of new cholinergic inhibitors from plant source is being conducted. For this purpose, the anti-AChE properties of *S. rhombifolia* were evaluated and reported for the first time in this study. Likewise, the non-polar extract, HEX extract (58.55%) possessed the strongest inhibition effect towards AChE, and followed by the EtOAc and MeOH extracts. By comparing with other medicinal plants, the plant extracts of *S. rhombifolia* are considered as strong cholinesterase inhibitors by giving 30–60% inhibition against AChE at the concentration of 100 μg/mL. Thirty-two medicinal plants were studied previously for AChE inhibition effects but only three plants, *Tabernaemontana divaricate*, *Stephania suberosa* and *Piper interruptum* showed higher inhibition than the HEX extract of *S. rhombifolia* at the same concentration tested (Ingkaninan et al. 2003). Furthermore, eight out of 10 medicinal plants studied by Ferreira et al. (2006) were not able to inhibit the enzyme up to a concentration of 500 μg/mL. Previous studies revealed that the cholinesterase inhibition effects of the plant extracts are mainly contributed by galanthamine and lycorine alkaloids, such as sanguinine and assoanine (Lopéz et al. 2002; Ingkaninan et al. 2003). The HEX extract, which gave the highest inhibition, is the most potential extract to be further studied on the discovery of lead compound through identification of the phytochemical constituents, thus identification of these constituents were carried out by GC-MS analysis in this study.

GC-MS analysis presented the volatile phytochemical constituents of the HEX extract of *S. rhombifolia*. The major constituents present are fatty acids, sterols and triterpenoids at various concentrations. The anti-inflammatory, anti-cholinergic and cytotoxic properties of the HEX extract are predicted to be contributed by these constituents. Two fatty acids, palmitic acid and linoleic acid were found in the HEX extract. The finding is agreed with a study reported by Bhatt et al. (1983). These compounds have been proven to show cytotoxic effects towards ascites tumor cells both *in vitro* and *in vivo* (Siegel et al. 1987). Besides, stigmasterol, sitosterol and lupeol are the steroids and triterpenoids present in the HEX extract. These constituents were previously isolated from this plant (Goyal & Rani 1988) and well known for their anti-inflammatory activities (Akihisa et al. 2000; Saleem 2009; Loizou et al. 2010). Apart from that, previous studies revealed the presence of alkaloids, such as ephedrine, phenethylamine, hypaphorine methyl ester, vasicine, and cryptolepinone (Prakash et al. 1981; Chaves et al. 2013). Other alkaloids might be present in the plant extracts and contribute to the observed cytotoxicity, as well as AChE inhibition effects (Lopéz et al. 2002; Ingkaninan et al. 2003; Chaves et al. 2013). Other classes of compounds that were reported to be found in the same genus of this plant are flavonoids, tocopherols, lignans, and coumarins (Jang et al. 2003; Silva et al. 2006; Chen et al. 2007). This suggests that the plant extracts of *S. rhombifolia* are rich in biologically active secondary metabolites, revealing that detailed phytochemical studies on this plant are worthwhile. Thus, further studies are strongly recommended to unveil the underlying mechanism and bioactive phytochemicals responsible for the observed activities in this study.

## Conclusions

The whole plant of *Sida rhombifolia* was extracted and evaluated for biological activities. The hexane extract possessed the strongest capabilities in anti-inflammation and anti-cholinesterase assay, as well as cytotoxicity against Hep G2 and SNU-1 cancer cell lines. Thus, the hexane extract of *S. rhombifolia* has a strong potential to be further developed into alternative medicine. Further works on the detail mechanism of the bioactive phytochemicals which contribute to the biological properties are strongly recommended.
